# *Salmonella* Enteritidis GalE Protein Inhibits LPS-Induced NLRP3 Inflammasome Activation

**DOI:** 10.3390/microorganisms10050911

**Published:** 2022-04-26

**Authors:** Tingting Huang, Dan Gu, Yaxin Guo, Ang Li, Xilong Kang, Xinan Jiao, Zhiming Pan

**Affiliations:** 1 Jiangsu Co-Innovation Center for Prevention and Control of Important Animal Infectious Diseases and Zoonoses, Yangzhou University, Yangzhou 225009, China; tingtingh309@163.com (T.H.); 006491@yzu.edu.cn (D.G.); gyxin220@163.com (Y.G.); lllang0510@163.com (A.L.); xlkang@yzu.edu.cn (X.K.); jiaoxa@yzu.edu.cn (X.J.); 2 Jiangsu Key Laboratory of Zoonosis, Yangzhou University, Yangzhou 225009, China; 3 Key Laboratory of Prevention and Control of Biological Hazard Factors (Animal Origin) for Agrifood Safety and Quality, Ministry of Agriculture of China, Yangzhou University, Yangzhou 225009, China; 4 Joint International Research Laboratory of Agriculture and Agri-Product Safety of the Ministry of Education, Yangzhou University, Yangzhou 225009, China

**Keywords:** *S.* Enteritidis, GalE, NLRP3 inflammasome, virulence, RNA-seq

## Abstract

Microbial infection can trigger the assembly of inflammasomes and promote secretion of cytokines, such as IL-1β and IL-18. It is well-known that *Salmonella* modulates the activation of NLRC4 (NLR family CARD domain-containing protein 4) and NLRP3 (NLR family pyrin domain-containing 3) inflammasomes, however the mechanisms whereby *Salmonella* avoids or delays inflammasome activation remain largely unknown. Therefore, we used *Salmonella* Enteritidis C50336Δ*fliC* transposon library to screen for genes involved in modulating inflammasomes activation. The screen revealed the galactose metabolism-related gene *galE* to be essential for inflammasome activation. Here, we found that inflammasome activation was significantly increased in J774A.1 cells or wild-type bone marrow-derived macrophages (BMDMs) during infection by Δ*fliC*Δ*galE* compared to cells infected with Δ*fliC*. Importantly, we found that secretion of IL-1β was Caspase-1-dependent, consistent with canonical NLRP3 inflammasome activation. Furthermore, the virulence of Δ*fliC*Δ*galE* was significantly decreased compared to Δ*fliC* in a mouse model. Finally, RNA-seq analysis showed that multiple signaling pathways related to the inflammasome were subject to regulation by GalE. Taken together, our results suggest that GalE plays an important role in the regulatory network of *Salmonella* evasion of inflammasome activation.

## 1. Introduction

*Salmonella* enterica subsp. enterica is a Gram-negative bacterium capable of infecting humans and other animals, and as such presents a threat to global public health [1]. Recently, *Salmonella* has been reported as the cause of approximately 9% of cases of diarrhea, and accounts for 41% of diarrhea-related deaths annually [2]. *Salmonella* is classified into more than 2600 serotypes based on surface antigenic composition [3,4]. *Salmonella* Enteritidis has been reported as one of the serotypes most significantly linked to gastroenteritis leading to persistent infection [5,6,7]. As a facultative intracellular pathogen, *S.* Enteritidis can invade and survive in both phagocytic and nonphagocytic cells, which may lower the treatment efficiency of traditional antibiotics and cause persistent infection in the host [8,9,10,11,12].

The innate immune system is critical for defense against invading pathogens and for mobilizing cellular pathways in the host that help identify and eradicate foreign bacteria [13]. The innate immune system recognizes the pathogen-associated molecular patterns (PAMPs) or damage-associated molecular patterns (DAMPs) via pattern recognition receptors (PRRs) [14]. The toll-like receptors (TLRs) and C-type lectin receptors (CLRs) recognize PAMPs and DAMPs localized to extracellular or vacuolar compartments [14]. The acid-inducible gene (RIG)-I-like, NOD-like receptors (NLRs), and AIM2-like receptors (ALRs) similarly sense cytoplasmic PAMPs and DAMPs [14]. When sensing PAMPs or DAMPs, the PRRs trigger the host immune system’s defense response by initiating any of a number of conserved signaling cascades. For example, the TLRs and CLRs can activate NF-κB and MAPK cascades to trigger the secretion of IL-6 and tumor necrosis factor alpha (TNF-α) as well as the expression of pro-IL-1β and pro-IL-18 [8,15,16]. Furthermore, the NLRs or ALRs promote the host defense response by encouraging the assembly of inflammasomes; the inflammasomes have, in turn, been shown to respond to *Salmonella* infection [17,18].

Inflammasomes are multi-protein complexes which are activated during *Salmonella* infection [19]. Inflammasomes consist of a PRR (such as NLR or ALR), an adapter protein ASC, and the cysteine protease Caspase-1 [20]. The PRRs recognize the type III secretion system (T3SS) needle protein, T3SS inner rod protein, and flagellin of pathogens, and upon recognition, activate Caspase-1 [21,22,23]. Activated Caspase-1 then cleaves the pro-inflammatory cytokines IL-1β and IL-18, as well as the pore-forming protein gasdermin D (GSDMD) [24,25]. GSDMD-N assembles to form pores in the plasma membrane of the host, leading to the release of active IL-1β and IL-18 [26]. In addition, the activation of Caspase-1 results in an inflammatory form of cell death called pyroptosis; subsequently, the bacteria are released to the extracellular environment from within the protected intracellular niche [26]. Released bacteria are taken up and killed by neutrophils, enhancing the clearance of intracellular pathogens [27].

*Salmonella* is transmitted via the fecal–oral route, and the immune system is activated during the invasion of intestinal epithelial cells by the bacteria; phagocytic cells are recruited to the site of infection as an inflammatory response is initiated [28]. *Salmonella* also triggers the activation of NLRP3 and NLRC4 inflammasomes [29]. The SPI-1 T3SS (PrgJ and PrgI) and flagellin proteins are recognized by the NLRC4 inflammasome, whereas the NLRP3 inflammasome recognizes host cell-derived stress and danger molecules and signals such as excessive potassium efflux, calcium influx, extracellular ATP, and mitochondrial reactive oxygen species, among others [30]. To evade clearance by the host innate immune system, *Salmonella* uses T3SS to inject effectors into host cells and downregulate the activation of inflammasomes, which promotes survival of *Salmonella* in the intracellular environment and leads to persistent infection [31,32]. *Salmonella* effector protein SopB inhibits the oligomerization of ASC and activation of the NLRC4 inflammasome to aid in immune escape [33]. By contrast, the effector proteins SipB and SopE induce the activation of Caspase-1 [34,35]. Interestingly, *Salmonella* employs SPI2 to subvert SPI1-induced NLRP3 and NLRC4 inflammasome activation in human primary macrophages, which is a species-specific immune evasion mechanism [36]. Collectively, these findings indicate that *Salmonella* regulates the activation of inflammasomes to evade clearance by the host immune system.

Previous studies have shown that FliC could strongly induce activation of NLRC4 inflammasome and downregulation of *fliC* could limit inflammasome activation [37,38]. Thus, we used the *S*. Enteritidis *fliC* deletion mutant strain which could reduce the activated level of inflammasome to screen for genes that inhibit the activation of inflammasomes in J774A.1 cells. In the present study, we identified the UDP-glucose 4-epimerase GalE protein, which inhibits the activation of Caspase-1 and is secreted into host cells. Infection of NLRP3- or NLRC4-deficient bone marrow-derived macrophages (BMDMs) with *Salmonella* revealed that inhibition of Caspase-1 by GalE was dependent on the NLRP3 inflammasome. Notably, RNA-seq analysis suggested that significantly enriched pathways were mainly related to the activation of inflammasomes during infection of macrophages with Δ*fliC*Δ*galE*. Collectively, our results provide important insight into the mechanisms whereby *S*. Enteritidis inhibits the activation of inflammasomes to evade clearance by the host immune system.

## 2. Materials and Methods

### 2.1. Bacterial Strains

The strains and plasmids used in this study are listed in Table S1. The targeted deletion strains were generated on the *S.* Enteritidis strain C50336 background in which the clean deletion mutant of *fliC* was constructed by double exchange of homologous recombination [39]. The transposon insertion library and deletion mutant strains were constructed on the C50336Δ*fliC* strain background. The upstream and downstream fragments of the *galE* gene were amplified by PCR using the primers listed in Table S2. The PCR products were inserted into the pDM4 plasmid using the ClonExpress MultiS One Step Cloning Kit (Vazyme, Nanjing, China). The recombinant plasmid was transformed into *E. coli* X7213 *λpir* cells, and clones were verified by PCR and sequencing. The plasmid Δ*galE*::pDM4 was transferred into C50336Δ*fliC* by conjugation, and the deletion mutant strain was screened on Luria-Bertani (LB) plates with 15% sucrose. The complementary strain Δ*fliC*Δ*galE*::*galE* was constructed with the plasmid pBAD33. The ORF of the *galE* gene was amplified by PCR and inserted into the pBAD33 plasmid using the ClonExpress II One Step Cloning Kit (Vazyme, Nanjing, China). The recombined plasmid *galE*::pBAD33 was transformed into Δ*fliC*Δ*galE*, and verified by PCR. The C50336Δ*fliC* -pCX340 and C50336Δ*fliC*-pCX340-*galE* strains were constructed for fluorescence resonance energy transfer (FRET) analysis. The PCR products of the *galE* gene were ligated into the pCX340 plasmid using *Nde* I and *Kpn* I restriction enzymes. The pCX340 or pCX340-*galE* constructs were electroporated into the wild type strain C50336Δ*fliC*.

### 2.2. Construction of Transposon Mutant Library and Transposon Insertion Sequencing

The donor strain *E. coli* X7213 *λpir* carrying plasmid pSC189 was grown on LB medium with DAP (50 μg/mL) and kanamycin (100 μg/mL), while the recipient strain C50336Δ*fliC* was cultured in LB medium. Equal volumes of donor bacteria and recipient bacteria suspensions were mixed and placed on the LB agar plate containing DAP and cultured at 37 °C for 12 h. The bacteria were washed in phosphate buffer (PBS) and inoculated on LB agar with kanamycin (100 μg/mL). The locations of transposon insertions were determined by two-round semi-arbitrary PCR, as previously described [40].

### 2.3. Mice and Cell Culture

Wild-type specific pathogen-free (SPF) female C57BL/6 mice were purchased from Vital River Laboratory Animal Technology Co., Ltd. (Beijing, China). The knock-out mice (*Casp1*^−/−^, *Nlrp3*^−/−^, and *Nlrc4*^−/−^) used in this study were bred on a C57BL/6 background (Shanghai Model Organisms Center, Inc., Shanghai, China), and all mice were bred under specific pathogen-free conditions. All mice were regularly fed clean and sterile rat chow and water and exposed to 12-h light/dark cycles; the temperature was maintained at 23 ± 1 °C. Animal experiments were approved by the Animal Welfare and Ethics Committees of Yangzhou University (SYXK[Su] 2017-0044) and were maintained in accordance with the guidelines of the Yangzhou University Institutional Animal Care and Use Committee (IACUC). All animals were healthy and in good spirits and were handled humanely to minimize distress throughout the course of experiments.

J774A.1 and HeLa cells were purchased from American Tissue Culture Collection (ATCC, Manassas, VA, USA), and cultured in DMEM with 10% (*v*/*v*) fetal bovine serum (FBS, Gibco) and 1% penicillin-streptomycin (GIBCO) at 37 °C in a 5% CO_2_ incubator. Wild-type, Nlrp3, Nlrc4, or Casp1-deficient bone marrow cells were flushed from the femurs and tibias of mice and cultured in DMEM supplemented with 10% (*v*/*v*) fetal bovine serum (FBS, Gibco), 1% penicillin-streptomycin (GIBCO), and 25 ng/mL macrophage colony-stimulating factor (M-CSF, PeproTech, Rocky Hill, NJ, USA) at 37 °C with 5% CO_2_ [41].

### 2.4. Salmonella Infection

Following overnight culture, bacteria were diluted into fresh LB medium at a concentration of 1:100 and incubated at 37 °C for 3.5 h. When necessary, arabinose was added at a final concentration of 0.04% (*g*/*v*) to induce the expression of GalE protein. The J774A.1 cells or different BMDMs were replated into 12-, 24-, or 96-well plates and cultured at 37 °C with 5% CO_2_ for 16–20 h before infection. For the activation of inflammasomes, monolayer cells were washed twice with Dulbecco’s PBS (DPBS), after which Opti-MEM medium with 1 μg/mL LPS was added (Sigma-Aldrich, St. Louis, MO, USA) and bacteria were cultured for 5 h. J774A.1 cells were infected with SE strains at a MOI of 100:1 while the BMDMs were infected with a MOI of 50:1; infected cells were incubated in Opti-MEM medium for 1.5 h. Subsequently, the cells were washed twice and incubated in Opti-MEM containing gentamicin (50 μg/mL) and cultured for another 3 h.

### 2.5. Adhesion and Invasion Assays

For adhesion assays, after infected J774A.1 cells for 30 min, the cultures were washed twice with PBS, and then incubated in PBS with Triton X-100 (0.2%) at 37 °C for 15 min, and the number of bacteria was determined by inspection of the LB agar culture medium. For invasion, the cells were washed with PBS after infection for 30 min and incubated in DMEM with 100 μg/mL gentamicin at 37 °C for 1 h. The number of bacteria was determined by inspection of the LB agar.

### 2.6. Cytotoxicity and Cytokine Analysis

BMDMs or J774A.1 cells were replated into 12-well plates at a density of 5 × 10^5^ per well and infected with SE strains as described above. At the indicated times, the supernatants were harvested for quantification of cytokine and LDH levels. LDH was measured using the LDH Cytotoxicity Assay Kit (Beyotime, Haimen, China) according to the manufacturer’s instructions. Supernatants were diluted as appropriate and release of IL-1β and IL-6 was evaluated by enzyme-linked immunosorbent assay (ELISA). Specific ELISA assays used in this study were the Mouse IL-1β/IL-1F2 DuoSet ELISA and Mouse IL-6 DuoSet ELISA (R&D Systems, Minneapolis, MN, USA), which were performed according to manufacturer instructions.

### 2.7. Western Blot Analysis

Protein samples from supernatants and cell lysates were prepared as previously described [18]. Three hours post-infection, supernatants were collected, and the cells were lysed in 300 μL cell lysis buffer for Western blot and IP (Beyotime, Heimen, China). The lysed cells were centrifuged to remove cell debris, and the supernatant was mixed with methanol and chloroform at a volume of 1:1:0.25. The mixed samples were centrifuged at 12,000 rpm for 5 min and the aqueous phase was discarded. Equal volumes of methanol were added to the samples, which were then vortexed vigorously and centrifuged at 12,000 rpm for 5 min. The pellets were dried at 55 °C for 5–10 min and resuspended in 40 μL 1× SDS-PAGE Sample Loading Buffer (Beyotime). Protein samples were resolved by SDS-PAGE using the standard 12% Tris–Glycine PAGE protocol. The protein was transferred to nitrocellulose membranes and subsequently blocked at room temperature for 1.5 h in PBS with 3% skim milk. Membranes were incubated with α-Caspase-1 p10 antibody (AdipoGen, San Diego, CA, USA), α-IL-1β antibody (Cell Signaling Technology, Danvers, MA, USA) or α-β-actin antibody (Sigma-Aldrich). The secondary antibody was goat anti-mouse IgG (Sigma-Aldrich). The membranes were visualized on an Amersham Imager 600 Imaging System (GE Healthcare, Pittsburgh, PA, USA) using ECL chemiluminescence substrate (Thermo Scientific, Waltham, MA, USA)

### 2.8. Fluorescence Resonance Energy Transfer (FRET)

Following overnight culture, SE strains Δ*fliC*-pCX340 and Δ*fliC*-pCX340-*galE* were diluted into fresh LB medium with 12.5 μg/mL tetracycline and cultured at 37 °C for 3.5 h. IPTG was added to induce the expression of GalE protein. The cultured bacteria were washed twice with DMEM and transfected into HeLa cells with an MOI of 100, and then centrifuged at 1000 rpm for 10 min. Three hours post-infection, the HeLa cells were washed with DMEM four times and infected for another 4 h in DMEM. The cell monolayers were washed twice with DMEM and loaded in 300 μL DMEM supplement (60 μL 6 × CCF2/AM) with a final concentration of 1 μM. The 6 × CCF2/AM was freshly prepared with the CCF2/AM loading kit (Invitrogen, Carlsbad, CA, USA). The cells were incubated at 37 °C for 2 h in the dark, and images were acquired using a Leica confocal microscope (Leica Microsystems, Wetzlar, Germany). The cells were excited at 405 nm, and emission at 450–470 nm and 520–540 nm resulted in either blue (cleaved) or green (uncleaved) fluorescence, respectively. Six hundred cells were randomly selected to calculate the percentage of GalE-positive cells.

### 2.9. RNA Sequencing Analysis

J774A.1 cells were seeded into 10-cm dishes at a density of 5 × 10^6^ cells and incubated overnight in DMEM containing 10% FBS. The cells were washed with PBS and pre-treated with LPS for 5 h. Cells were infected with Δ*fliC* and Δ*fliC*Δ*galE* at a MOI of 100:1 and cultured in DMEM for 4 h. The supernatants were removed, and cell lysates were collected in 3 mL Trizol (Ambion, Carlsbad, CA, USA) to extract total RNA. The three parallel RNA isolates were sequenced by illumine Hi-Seq (GENEWIZ, Suzhou, China), and the following analyses were performed as previously described [42]. Briefly, the raw data were quantified and filtered by Cutadapt (version 1.9.1) [43], and then mapped to the reference genome Mus_musculus_GRCm39.104_Ensembl using the HISAT2 software package (version 2.0.1) [44]. The transcript levels of the genes were estimated by HTSEQ (version 0.6.1) [45], and differential expression analysis was conducted by DESeq2 (version 1.26.0) [46].

### 2.10. Quantitative Real-Time PCR Analysis

J774A.1 cells were infected with Δ*fliC* and Δ*fliC*Δ*galE* strains as described above. The total RNA was extracted using the FastPure Cell/Tissue Total RNA Isolation Kit (Vazyme, Nanjing, China). Isolated total RNA was about 700–800 ng/μL, and the purity (A260/280 = 2.04~2.1, A260/230 = 2.11~2.2) and integrity (RIN = 9.7~10) were within the normal range. Equal quantities of total RNA were reversed-transcribed to cDNA using Hiscript III RT Super mix (Vazyme, Nanjing, China) according to the manufacturer’s instructions. Real-Time PCR was performed using the Universal SYBR qPCR Master Mix Kit (Vazyme, Nanjing, China) in a 20 μL reaction volume containing 200 ng of cDNA, 10 μL of SYBR qPCR Master mix, 0.6 μL primers, and 6.8 μL RNase-free water. The qRT-PCR reaction was performed using an Applied Biosystems QuantStudio 6 Flex RealTime PCR System (Applied Biosystems, Foster City, CA, USA) using the following protocol: 95 °C for 10 min, 40 cycles at 95 °C for 15 s, and 60 °C for 1 min. The relative expression levels of genes were calculated using the comparative threshold cycle (2^−ΔΔCT^) method. The qRT-PCR experiments were performed in triplicates.

### 2.11. In Vivo Virulence Assay

Six-week-old female C57BL/6 mice were randomly assigned to three groups (*n* = 8). Bacteria were grown to logarithmic phase, washed twice with PBS, and resuspended in PBS. Each group was infected with 200 μL Δ*fliC* or Δ*fliC*Δ*galE* diluted in PBS at a dose of 5 × 10^6^ CFU per mouse. The control mice received 200 μL of PBS under otherwise identical conditions. Bodyweight and mortalities were monitored and recorded for all mice over 14 dpc.

### 2.12. Statistical Analysis

A standard Z score was used to estimate the cytotoxicity of each insertion mutant strain as previously described [47]. Statistical significance was defined as a Z score ≤ −2 or ≥2. Statistical analyses were performed using GraphPad Prism 8.0 software. All results were expressed as the mean ± SEM. Statistical significance was assigned at *p* values of <0.05 (*), <0.01(**), or <0.001 (***) based on the Student’s *t*-test.

## 3. Results

### 3.1. Screening Candidate Salmonella Genes That Modulate Inflammasome Activation

**Figure 1:**
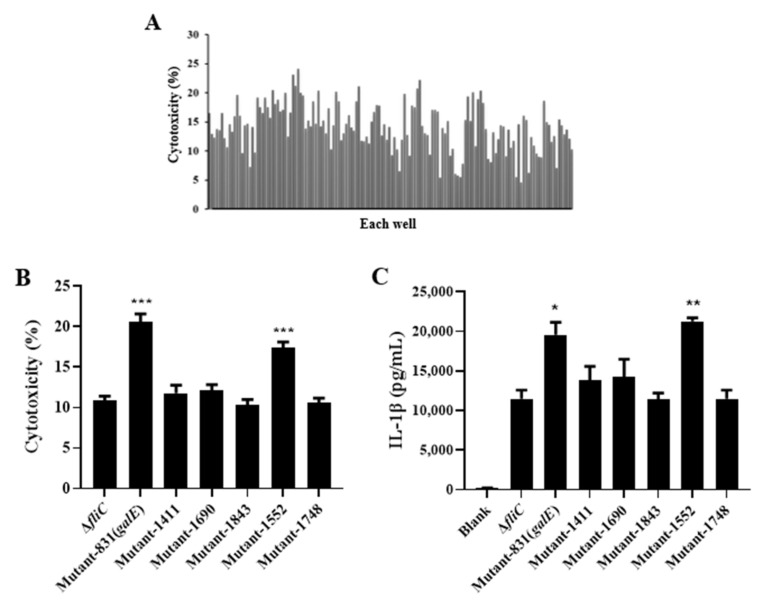

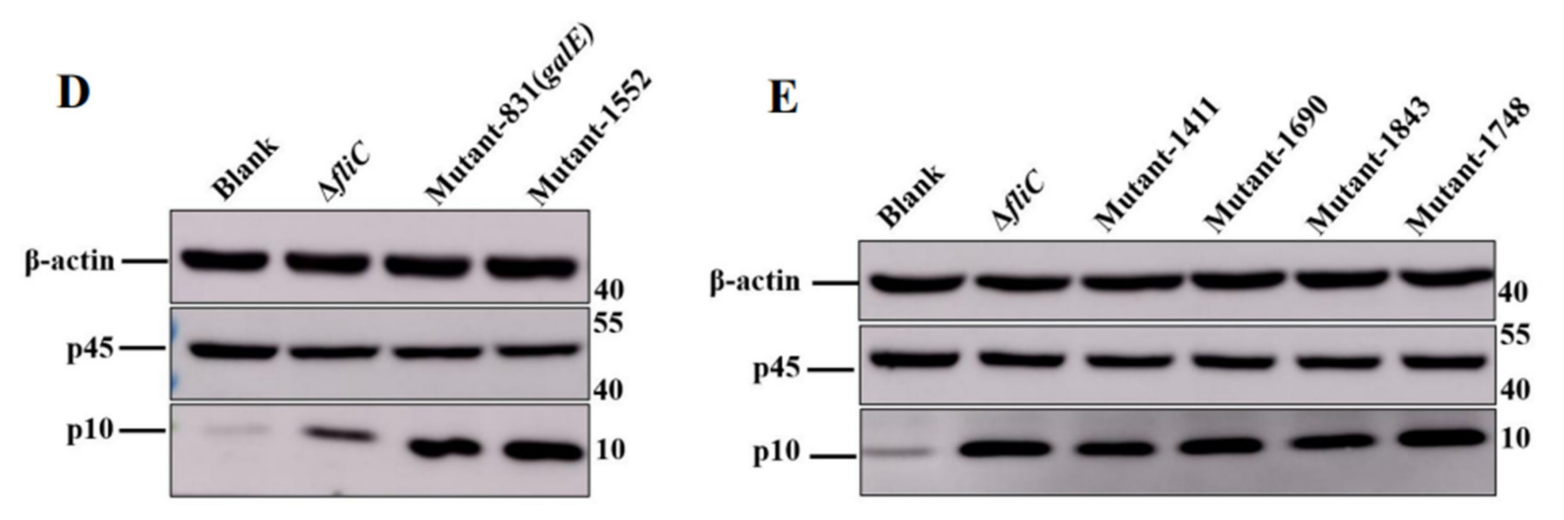
Identification of genes involved in modulating inflammasome activation in vitro. (**A**) J774A.1 cells were pre-treated with LPS (1 μg/mL, 5 h) and then infected with C50336Δ*fliC* TnpSC189 transposon insertion mutants at an MOI of 100 for 4 h. The release of LDH was measured in supernatants of infected cells for the first-round screen. (**B**) Release of LDH in supernatants of J774A.1 cells infected with candidate mutants. (**C**) Levels of IL-1β in culture were determined via ELISA. (**D**,**E**) Activation of Caspase-1 (p10) was assessed by Western blot, and β-actin was used as a loading control. Molecular mass markers (in kDa) are indicated on the right. Data are presented as mean ± SEM of three independent experiments, * *p* < 0.05, ** *p* < 0.01, *** *p* < 0.001.

Pathogens can modulate the activation of inflammasomes to evade their clearance by the host immune system. Therefore, we generated a transposon insertion library in a flagellin (*fliC*) deletion mutant strain of *S.* Enteritidis C50036 to screen for genes that modulate inflammasome activation. The mutants were first screened based on whether we observed an increase in Lactate dehydrogenase (LDH) release relative to levels observed in the C50336Δ*fliC* parental strain 4 h after infection of J774A.1 cells (Figure 1A). Z-scores were calculated for the insertion mutants, and differences were deemed significant if the Z score was ≥2 or ≤−2 (Figure S1A). The candidate mutants were further evaluated for cytotoxicity (Figure 1B), activation of Caspase-1 (Figure 1D–E), and secretion of IL-1β (Figure 1C). These results showed that the candidate mutants 831 and 1552 significantly enhanced cytotoxicity, Caspase-1 activation, and IL-1β induction. Intriguingly, sequencing the transposon insertions of candidate mutants identified the gene *galE* (Figure S1B). The *galE* gene encodes UDP-glucose 4-epimerase, which catalyzes epimerization between UDP-glucose (UDP-Glc) and UDP-galactose (UDP-Gal). However, the potential role of GalE in modulating inflammasome activation is unknown.

### 3.2. S. Enteritidis GalE Inhibits the Activation of Inflammasomes

**Figure 2:**
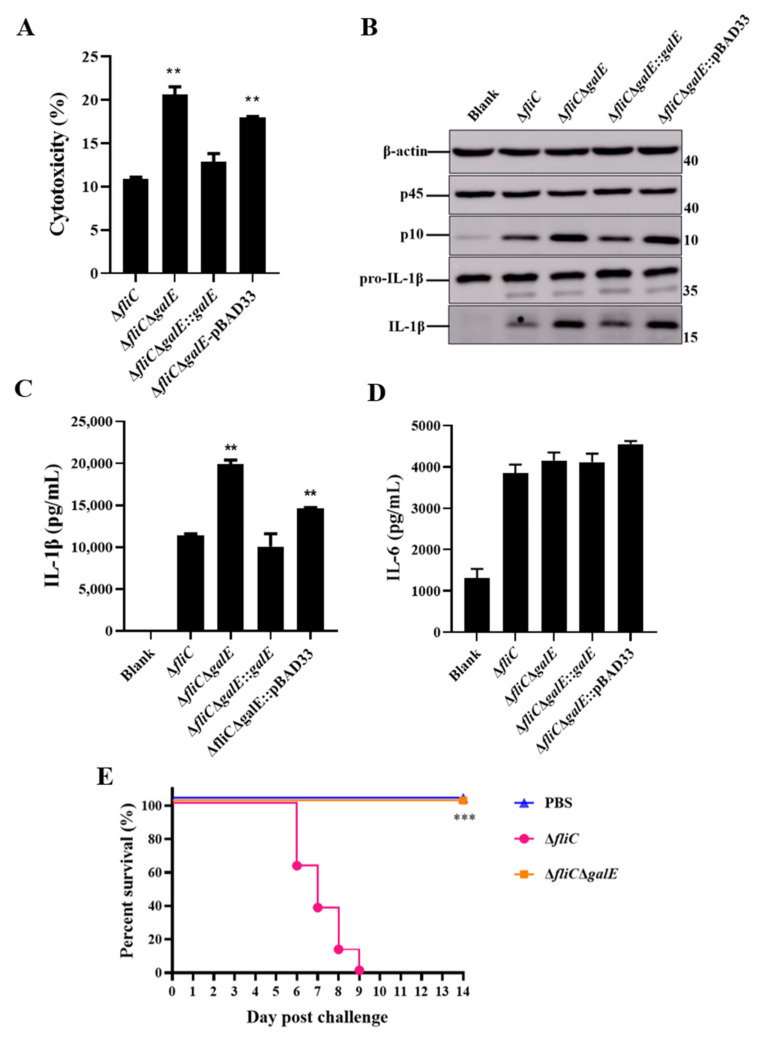
*Salmonella* GalE protein inhibits the activation of inflammasomes. J774A.1 cells were pre-treated with LPS (1 μg/mL, 5 h) and then infected with WT strain Δ*fliC*, Δ*fliC*Δ*galE*, *galE*-complemented Δ*fliC*Δ*galE*::*galE*, or empty vector-complemented Δ*fliC*Δ*galE*::pBAD33 at an MOI of 100 for 4 h. Arabinose (0.04%, *g*/*v*) was added to induce the expression of GalE protein. Uninfected cells were used as a negative control (Blank). (**A**) Release of LDH in supernatants of infected cells. (**B**) Activation of Caspase-1 and secretion of IL-1β were analyzed by Western blot. C-D. ELISA for IL-1β (**C**) and IL-6 (**D**). Data are presented as mean ± SEM of three independent experiments, ** *p* < 0.01, *** *p* < 0.001. (**E**) C57BL/6 mice were orally infected with 5 × 10^6^ CFU of Δ*fliC* or Δ*fliC*Δ*galE* mutant strains and survival of the mice was monitored over 14 d. Bacteria-free PBS was administered to a separate group of animals as a negative control.

**Figure 3:**
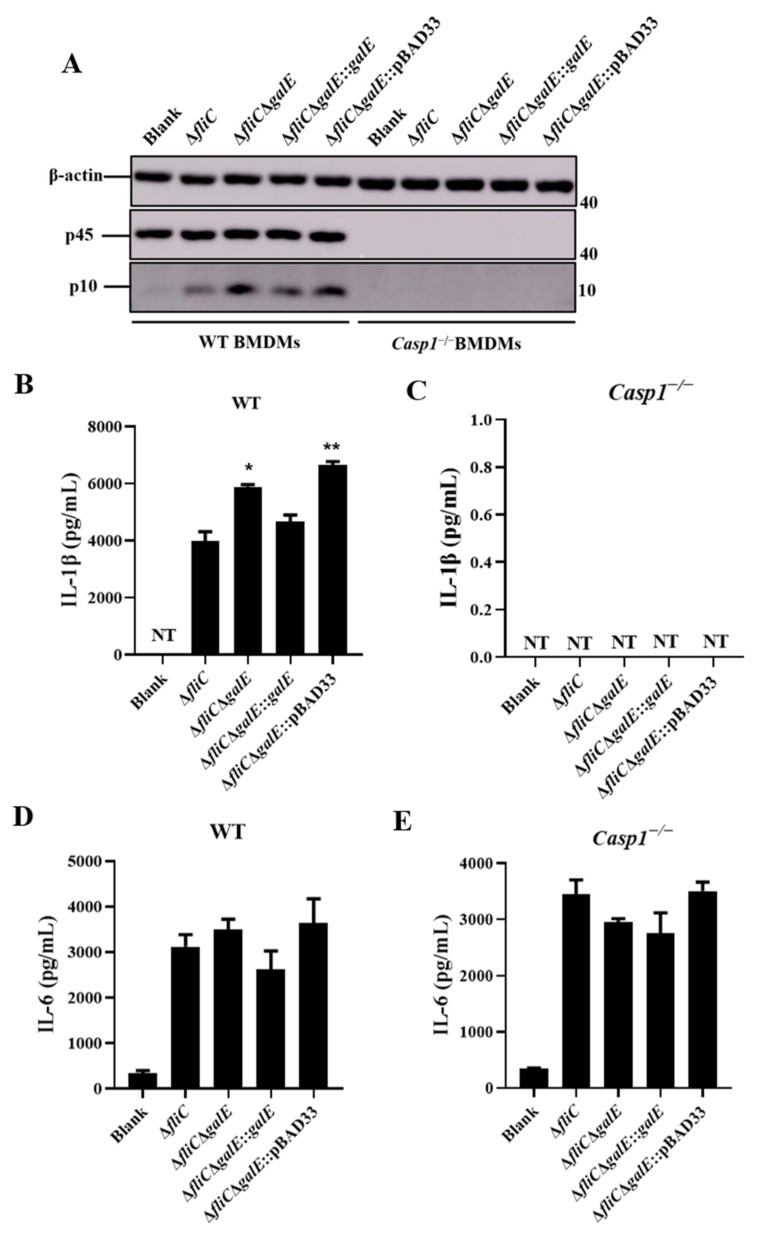
GalE inhibits Caspase-1-dependent inflammasome activation. BMDMs from WT and *Casp1*^−/−^ mice were primed with LPS (100 ng/mL, 5 h) and then infected with WT strain Δ*fliC*, Δ*fliC*Δ*galE*, *galE*-complemented Δ*fliC*Δ*galE*::*galE*, or empty vector-complemented Δ*fliC*Δ*galE*::pBAD33 for the indicated time periods at an MOI of 50 for 4 h. (**A**) Activation of Caspase-1 analyzed by immunoblotting. (**B**,**C**) ELISA for IL-1β in supernatants of WT BMDMs (**B**) or *Casp1*^−/−^ BMDMs (**C**). (**D**,**E**). ELISA for IL-6 in supernatants of WT BMDMs (**D**) or *Casp1*^−/−^ BMDMs. (**E**) Data are presented as mean ± SEM of three independent experiments, * *p* < 0.05, ** *p* < 0.01.

To further investigate the role of GalE in inflammasome activation, we constructed a *galE* in-frame deletion mutant and complementary strain. Growth curve results indicated that there was no significant difference among the Δ*fliC*, Δ*fliC*Δ*galE*, and complementary strains (Figure S2A). We next evaluated these strains for their ability to activate inflammasomes. As expected, J774A.1 cells infected with Δ*fliC*ΔgalE and empty plasmid complementary Δ*fliC*Δ*galE*::pBAD33 strains produced significantly higher levels of LDH (Figure 2A) and exhibited greater Caspase-1 activation (Figure 2B) in comparison with J774A.1 cells infected by the parental strain Δ*fliC*; levels observed in the complementary strain were restored to the levels observed in cells infected with Δ*fliC*. Furthermore, the Δ*fliC*Δ*galE* and Δ*fliC*Δ*galE*::pBAD33 strains induced significant Caspase-1-dependent cytokine IL-1β secretion in J774A.1 cells (Figure 2C), while the inflammasome-independent cytokine IL-6 was not affected (Figure 2D). Meanwhile, the activation of Caspase-1 (Figure 3A) and release of IL-1β (Figure 3B,C) was inhibited in the *Casp1*^−/−^ deficient BMDMs, while the release of IL-6 was not affected in the *Casp1*^−/−^ deficient BMDMs (Figure 3D,E), suggesting the secretion of IL-1β was Caspase-1-dependent. Taken together, these results suggest that the GalE protein inhibits activation of inflammasomes.

To investigate whether the increased activation of inflammasomes caused by Δ*fliC*Δ*galE* was due to reduced ingestion of intracellular bacteria by macrophages, we assessed the adhesion and invasion capabilities of *S*. Enteritidis in J774A.1 cells. The results indicated that the adhesion and invasion of J774A.1 cells were unaffected by GalE (Figure S2B,C), indicating that induction of inflammasome activation by Δ*fliC*Δ*galE* is independent of bacterial adherence-invasion rates. We used C57BL/6 mice as a model in which to test the impact of GalE on the virulence of *S*. Enteritidis in vivo. Strikingly, all mice infected with Δ*fliC* died within 9 days, whereas none of the mice in the Δ*fliC*Δ*galE*-infected group died (Figure 2E). Collectively, these results suggest that inhibition of inflammasome activation by GalE greatly increases the virulence of *S*. Enteritidis.

### 3.3. S. Enteritidis GalE Inhibits the Activation of Caspase-1 in Macrophages via the NLRP3 Inflammasome

**Figure 4:**
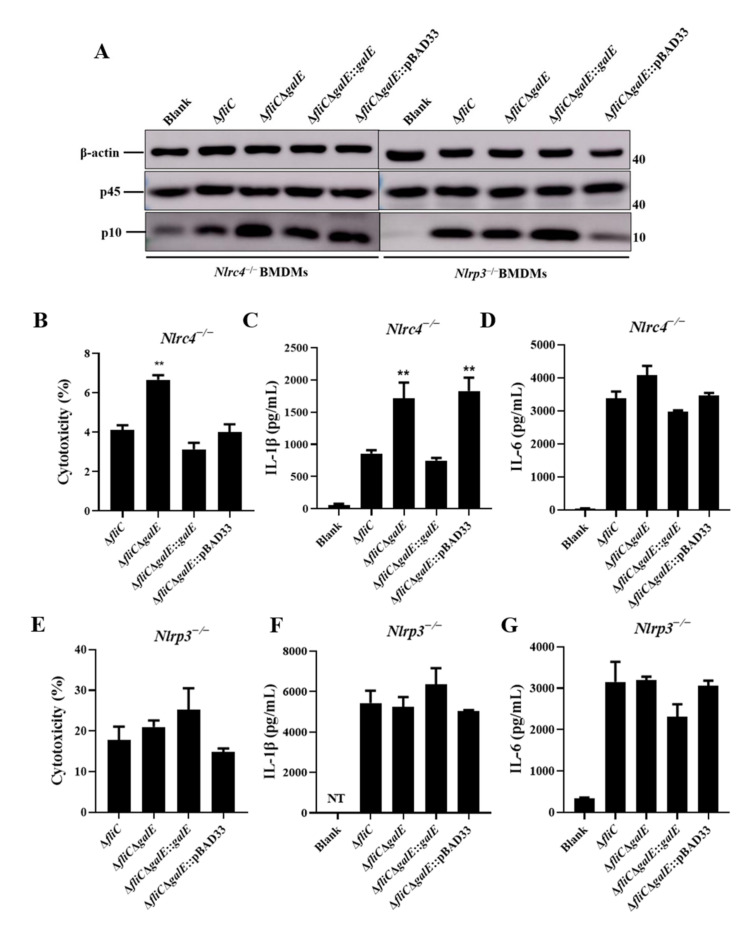
GalE inhibits NLRP3-dependent inflammasome activation. BMDMs from *Nlrc4*^−/−^ or *Nlrc3*^−/−^ mice were primed with LPS (100 ng/mL, 5 h) and then infected with WT strain Δ*fliC*, Δ*fliC*Δ*galE*, *galE*-complemented Δ*fliC*Δ*galE*::*galE*, or empty vector-complemented Δ*fliC*Δ*galE*:: pBAD33 for the indicated time periods at an MOI of 50 for 4 h. (**A**) Activation of Caspase-1 was analyzed by immunoblotting. (**B**–**D**) Levels of LDH (**B**), IL-1β (**C**), and IL-6 (**D**) in supernatants of *Nlrc4*^−/−^ BMDMs. (**E**–**G**). Levels of LDH (**E**), IL-1β (**F**), and IL-6 (**G**) in supernatants of *Nlrp3*^−/−^ BMDMs. Data are presented as mean ± SEM of three independent experiments, ** *p* < 0.01.

It is well-known that the NLR family members NLRP3 and NLRC4 are recognized by *Salmonella* and mediate the activation of Caspase-1 [48]. To further investigate the mechanisms responsible for Caspase-1 activation by *S*. Enteritidis GalE protein, we monitored the activation of Caspase-1 in *Nlrp3*^−/−^ and *Nlrc4*^−/−^-deficient BMDMs in response to infection with different mutant strains of *S*. Enteritidis. Caspase-1 activation was significantly increased in *Nlrc4*^−/−^ BMDMs during infection with Δ*fliC*Δ*galE* and Δ*fliC*Δ*galE*::pBAD33 strains compared to Δ*fliC*, but no such differences were observed in *Nlrp3*^−/−^ BMDMs infected with different mutant strains (Figure 4A). Similarly, the release of LDH and IL-1β were increased in the *Nlrc4*^−/−^ BMDMs infected with the Δ*fliC*Δ*galE* strain relative to BMDMs infected by the parental Δ*fliC* strain (Figure 4B,C), and no differences were observed in *Nlrp3*^−/−^ BMDMs (Figure 4E,F). Furthermore, the level of non-inflammasome cytokine IL-6 was unaffected in Δ*fliC*Δ*galE*-infected *Nlrc4*^−/−^ and Nlrp3-BMDMs compared to levels observed in wild-type Δ*fliC*-infected cells (Figure 4D,G). Taken together, these results indicate that the GalE protein inhibits the activation of Caspase-1 via an NLRP3-dependent canonical inflammasome activation pathway.

### 3.4. GalE Protein Is Secreted in HeLa Cells

**Figure 5:**
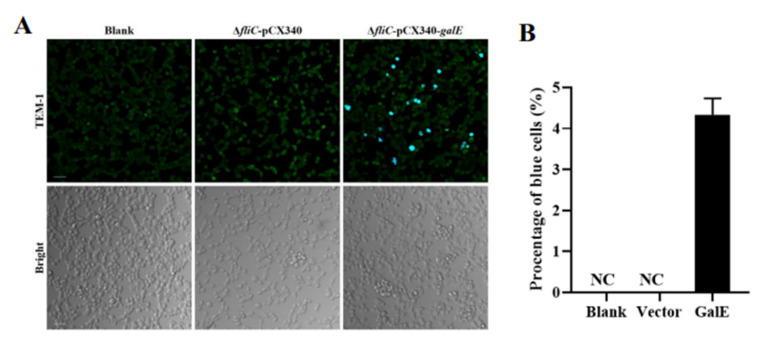
GalE is secreted in HeLa cells. (**A**) HeLa cells were infected with *S*. Enteritidis Δ*fliC* strains carrying pCX340 or pCX340-*galE*. Translocation of TEM-1-GalE fusion protein into the HeLa cells results in cleavage of CCF2-AM and emission of blue fluorescence, whereas uncleaved CCF2-AM emits green fluorescence. Scale bar = 50 μm. (**B**) Blue fluorescent cells expressed as a percentage of total cells. For each cell well, three pictures were taken and approximately 600 cells were counted to calculate the percentage of blue fluorescent cells. Data are presented as Mean ± SD of triplicate samples.

*Salmonella* secretes effector proteins into host cells to influence the activation of inflammasomes [49]. To assess the secretion of GalE into host cells, we took advantage of fluorescence resonance energy transfer (FRET). Here, β-lactamase TEM-1 was infused with *galE*, and intracellular translocation was assayed via FRET in infected HeLa cells. As shown in Figure 5A, blue fluorescent cells were observed during infection with Δ*fliC* containing the pCX340-*galE* plasmid, while no blue fluorescence was observed in untreated HeLa cells and HeLa cells infected with the Δ*fliC*-pCX340 strain. More than 600 cells were randomly selected to calculate the percentage of positive cells; the transfer efficiency of GalE-TEM was estimated to be about 4.3% (Figure 5B). These results indicate that the GalE protein is secreted into host cells during infection.

### 3.5. Identification of Inflammasome Activation Pathways Inhibited by the GalE Protein

**Figure 6:**
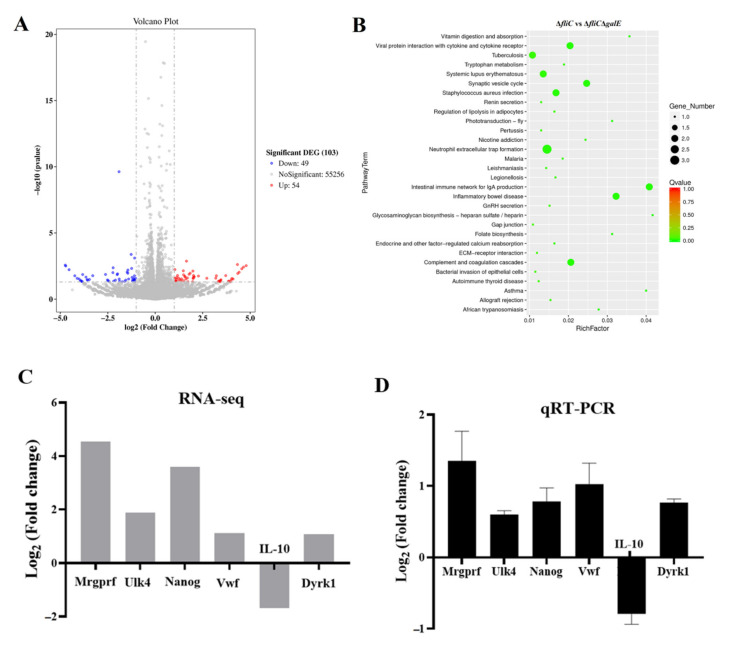
Identification of genes involved in GalE-dependent modulation of inflammasomes. J774A.1 cells were pre-treated with LPS (1 μg/mL, 5 h) and then infected with Δ*fliC* and Δ*fliC*Δ*galE* at an MOI of 100 for 4 h. The cells were collected, and the total RNA was isolated for RNA sequencing or for qRT-PCR validation of differentially expressed genes (DEGs). (**A**) Volcano plot for the transcription profiles of J774A.1 cells infected with Δ*fliC* and Δ*fliC*Δ*galE*. Numbers of DEGs are presented on the righthand side of the volcano plot. Blue dots represent downregulated genes and red dots represent upregulated genes. (**B**) KEGG enrichment analysis of DEGs between the Δ*fliC*-infected group and the Δ*fliC*Δ*galE*-infected group. (**C**) DEGs in infected J774A.1 cells as identified by RNA-seq. (**D**) qRT-PCR was used to validate DEGs. mRNA expression levels were normalized to GAPDH. Data are presented as mean ± SEM of triplicate independent experiments.

Transcriptome analyses of J774A.1 cells infected with Δ*fliC* and Δ*fliC*Δ*galE* were performed to identify the pathways involved in regulating the activation of inflammasomes via the *S*. Enteritidis GalE protein. Differentially expressed genes were chosen according to criteria of a fold change value |log2-Ratio| ≥ 1 and false discovery rate (*P*-adjust) < 0.05. Expression of a total of 10^3^ genes was determined to differ significantly between the Δ*fliC*-infected and Δ*fliC*Δ*galE*-infected groups, including 54 up-regulated genes and 49 down-regulated genes (Figure 6A). The Kyoto Encyclopedia of Genes and Genomes (KEGG) signaling pathway analysis showed that these differentially expressed genes were enriched in inflammatory bowel disease, neutrophil extracellular trap formation, systemic lupus erythematosus, and an intestinal immune network for IgA production (Figure 6B) and were primarily related to inflammation. Six differentially expressed genes in infected J774A.1 cells were selected for verification by quantitative real-time PCR. Mouse glyceraldehyde-3-phosphate dehydrogenase (GAPDH) was used as a control gene. The expression levels of these genes were consistent with the results of RNA-seq in J774A.1 cells infected with Δ*fliC*Δ*galE* when compared to the cells infected with Δ*fliC* (Figure 6C,D). These results demonstrate that *galE* plays an important role in regulating inflammation-related pathways.

## 4. Discussion

To evade clearance by the host immune system, pathogens have developed multiple strategies to manipulate host cell physiology and survive in these cells. Previous studies have reported that the activation of inflammasomes is an important process whereby pathogens evade immune system clearance [50]. The pathogenic *Yersinia* species possesses several effector proteins that modulate the activation of inflammasomes and facilitate bacterial colonization of the host [51]. As a facultative intracellular pathogen, *Salmonella* also secretes effector proteins into host cells to modulate the activation of inflammasomes [52]. Thus, we screened genes involved in inhibiting the activation of inflammasomes using an *S*. Enteritidis Δ*fliC* transposon insertion library and found that the GalE protein is capable of inhibiting inflammasome activation. We further found that the GalE protein can be secreted into host cells and inhibits the activation of Caspase-1 in an NLRP3-dependent way.

GalE is a UDP-galactose-4-epimerase which catalyzes the interconversion between UDP-glucose (UDP-Glc) and UDP-galactose (UDP-Gal), an essential process in sugar metabolism. GalE mediates the incorporation of galactose into the O-side chain of lipopolysaccharide (LPS), which is an important protective antigen for *Salmonella*. GalE is also essential for virulence in many pathogens, including *Salmonella*, *Pasteurella multocida*, and *Campylobacter jejuni* [53,54,55]. Previous studies have indicated that the virulence of *Salmonella* typhimurium and *Salmonella* typhi *galE* deletion mutant strains was significantly reduced, and these mutant strains could be developed as a live oral vaccine [55,56]. Our results revealed that the *S*. Enteritidis *galE* deletion mutant strain also possessed attenuated virulence in C57BL/6 mice (Figure 2E), while the lack of the *galE* gene did not impair bacterial adhesion and invasion ability in J774A.1 cells (Figure S2B,C). These results imply that the GalE protein is essential for the virulence of *S*. Enteritidis.

*Salmonella* flagellin and SPI-1 PrgJ/PrgI are recognized by NLRC4, leading to the rapid activation of the inflammasome, whereas the NLRP3 inflammasome responds to diverse structures of both host and foreign origin [57]. Inflammasome activation can facilitate the host defense against *Salmonella* infection [27]. However, *Salmonella* can also inhibit the activation of inflammasomes and allow the bacteria to evade host immune system clearance [50,58]. Here, we demonstrated that the Δ*fliC*Δ*galE* strain stimulates increased LDH release, increased Caspase-1 activation, and increased IL-1β secretion both in wild-type BMDMs and J774A.1 cells relative to cells infected with the Δ*fliC* strain. Interestingly, Δ*fliC*Δ*galE* significantly induced Caspase-1 activation, IL-1β secretion, and release of LDH compared to the *Nlrc4*^−/−^ BMDMs infected with Δ*fliC* strain, indicating that GalE inhibit the activation of Caspase-1 was independent of NLRC4. However, no significant difference was observed in *Nlrp3*^−/−^ BMDMs infected with Δ*fliC*Δ*galE* or Δ*fliC*, suggesting that GalE inhibit the activation of Caspase-1 was dependent of NLRP3. Caspase-1-activated signals were not detected in *Casp1*^−/−^ BMDMs. These findings suggest that GalE plays an important role in the process of *Salmonella*’s evasion of clearance by the host inflammasome.

Interestingly, *Salmonella* can modulate the activation of inflammasomes by disrupting glycolysis to invade macrophages. Decreased levels of NADH and induction of mitochondrial ROS products resulting from these disruptions in the glycolytic pathway can in turn induce NLRP3 inflammasome activation [59]. Consistent with these observations, the *Salmonella* TCA enzyme aconitase was identified as essential for long-term persistent *Salmonella* infection in a transposon-based genome-wide screen [60]. The absence of aconitase induces the rapid activation of the NLRP3 inflammasome, which is associated with elevated levels of the TCA metabolite citrate and mitochondrial ROS products [61]. Therefore, *Salmonella* infection disrupts host cell metabolism, which in turn triggers activation of the NLRP3 inflammasome. Here, we described another *Salmonella* gene, *galE*, and demonstrated that deletion of the *galE* gene could significantly induce NLRP3 inflammasome activation. The absence of the *Salmonella galE* gene results in the use of exogenous galactose in the host cell which may disrupts metabolism [62,63]. Thus, our results further confirmed that the disruption of host cell metabolism may modulate inflammasome activation. In addition, *Salmonella* preferentially associates with anti-inflammatory/M2 macrophages which are capable of utilizing fatty acid metabolites rather than glycolysis at the later stages of infection to avoid triggering an inflammatory response [64]. Our work and previous studies collectively suggest that metabolites may serve as molecular signals to the innate immune cells to initiate the recruitment of inflammasomes and secretion of cytokines.

We also identified differentially expressed genes and enriched pathways in *Salmonella* infected macrophages. This analysis uncovered multiple signaling pathways that are associated with inflammasome activation and inflammation. Inflammation-associated molecules have been shown elsewhere to stimulate the synthesis and release of von Willebrand Factor (vWF), a biomarker of inflammation [65]. Dyrk1b is an arginine-directed serine/threonine protein kinase which is highly expressed in many cancers. Increased levels of Dyrk1b have been found to interact with STAT3 to modulate the activation of astrocyte cells in LPS-induced neuroinflammation [66]. Furthermore, ULK4 has been associated with primary biliary cholangitis (PBC); expression levels of ULK4 were found to be significantly higher in PBC patients than in healthy controls, suggesting a potential relationship between ULK4 and inflammation [67]. Our expression analyses showed that transcript levels of *vwf*, *dyrk1b*, and *ulk4* were significantly elevated in macrophages infected with the Δ*fliC*Δ*galE* strain (Figure 6C,D), indicating that the pathways regulated by these genes may also play a role in GalE protein regulation and its contribution to inflammasome activation. Besides, ULK4 has been reported as an autophagy-related gene and acts upstream of PIK3C3 to regulate the formation of autophagophore [68]. The previous studies have also reported that the autophagy can cause excessive activation of NLRP3 inflammasome [69]. Thus, we may speculate that the ULK4 involved in the activation of inflammasome is dependent on the autophagy. Interleukin-10 (IL-10) is a key anti-inflammatory cytokine secreted by activated immune cells [70]. Furthermore, IL-10 can inhibit the secretion of IL-1β by reducing pro-IL-1β protein or inhibiting the activation of the NLRP3 inflammasome [71]. In our study, we observed that expression levels of IL-10 were suppressed in J774A.1 cells infected with the Δ*fliC*Δ*galE* strain (Figure 6C,D), which could explain why Δ*fliC*Δ*galE* significantly enhanced inflammasome activation in infected cells.

## 5. Conclusions

In this study, we first established that *S*. Enteritidis GalE protein inhibits the activation of the NLRP3 inflammasome in infected cells and is essential for the virulence of *S*. Enteritidis. Furthermore, we found that the GalE protein can be secreted into host cells to modulate inflammasome activation. Our RNA-seq data indicated that multiple signaling pathways were enriched in infected cells, and that these pathways were primarily related to inflammasome activation. We demonstrated that the GalE protein inhibits activation of the NLRP3 inflammasome to promote the virulence of *S*. Enteritidis. Collectively, our data provide evidence that *Salmonella* disrupts the homeostasis of metabolism in host cells to inhibit the activation of inflammasomes, offering a putative mechanism of bacterial immune evasion strategies.

## Data Availability

The raw sequence data of RNA-seq were deposited in NCBI with the accession number PRJNA820156.

## Supplementary Materials

The following supporting information can be downloaded at: https://www.mdpi.com/article/10.3390/microorganisms10050911/s1, Table S1: Bacterial strains and plasmids used in this study. Table S2: The primers used in this study. Figure S1: Analysis of candidate transposon insertion mutants. (A) Z scores were calculated for each individual well in a 48-well cell plate, and a Z score ≤ −2 or ≥2 was considered significant. (B) Insertion sites of transposon mutants. Horizontal arrows indicate the direction of gene expression. The green arrows represent candidate genes; blue arrows represent the upstream gene, and brown arrows represent the downstream gene. Numbers represent the initial or terminal position of these genes in the *Salmonella* Enteritidis genome. Red vertical arrows indicate the transposon insertion site of the candidate transposon mutant. Figure S2: Functional characterization of the GalE protein. (A) The growth curves of *S*. Enteritidis Δ*fliC*, Δ*fliC*Δ*galE*, Δ*fliC*Δ*galE*::*galE*, and Δ*fliC*Δ*galE*:: pBAD33. (B,C) The adhesion (B) and invasion (C) ability of S. Enteritidis Δ*fliC*, Δ*fliC*Δ*galE*, Δ*fliC*Δ*galE*::*galE*, and Δ*fliC*Δ*galE*::pBAD33 in J774A.1 cells. References [18,72,73,74,75,76] cited in Supplementary Materials.

## References

[1] Majowicz S.E., Musto J., Scallan E., Angulo F.J., Kirk M., O’Brien S.J., Jones T.F., Fazil A., Hoekstra R.M., Int Collaboration Enteric Dis B. (2010). The Global Burden of Nontyphoidal *Salmonella* Gastroenteritis. Clin. Infect. Dis..

[2] Besser J.M. (2018). *Salmonella* epidemiology: A whirlwind of change. Food Microbiol..

[3] Brenner F.W., Villar R.G., Angulo F.J., Tauxe R., Swaminathan B. (2000). *Salmonella* nomenclature—Guest commentary. J. Clin. Microbiol..

[4] Popoff M.Y., Bockemuhl J., Brenner F.W. (2000). Supplement 1998 (no. 42) to the Kauffmann-White scheme. Res. Microbiol..

[5] Ferrari R.G., Rosario D.K.A., Cunha-Neto A., Mano S.B., Figueiredo E.E.S., Conte-Junior C.A. (2019). Worldwide Epidemiology of *Salmonella* Serovars in Animal-Based Foods: A Meta-analysis. Appl. Environ. Microbiol..

[6] Sanchez-Vargas F.M., Abu-El-Haija M.A., Gomez-Duarte O.G. (2011). *Salmonella* infections: An update on epidemiology, management, and prevention. Travel Med. Infect. Dis..

[7] Eguale T., Gebreyes W.A., Asrat D., Alemayehu H., Gunn J.S., Engidawork E. (2015). Non-typhoidal *Salmonella* serotypes, antimicrobial resistance and co-infection with parasites among patients with diarrhea and other gastrointestinal complaints in Addis Ababa, Ethiopia. BMC Infect. Dis..

[8] Haraga A., Ohlson M.B., Miller S.I. (2008). *Salmonella* e interplay with host cells. Nat. Rev. Microbiol..

[9] Niedergang F., Sirard J.C., Blanc C.T., Kraehenbuhl J.P. (2000). Entry and survival of *Salmonella* typhimurium in dendritic cells and presentation of recombinant antigens do not require macrophage-specific virulence factors. Proc. Natl. Acad. Sci. USA.

[10] Stapels D.A.C., Hill P.W.S., Westermann A.J., Fisher R.A., Thurston T.L., Saliba A.-E., Blommestein I., Vogel J., Helaine S. (2018). *Salmonella* persisters undermine host immune defenses during antibiotic treatment. Science.

[11] Tang Y., Foster N., Jones M.A., Barrow P.A. (2018). Model of Persistent *Salmonella* Infection: *Salmonella* enterica Serovar Pullorum Modulates the Immune Response of the Chicken from a Th17-Type Response towards a Th2-Type Response. Infect. Immun..

[12] Mon K.K.Z., Kern C., Chanthavixay G., Wang Y., Zhou H. (2021). Tolerogenic Immunoregulation towards *Salmonella* Enteritidis Contributes to Colonization Persistence in Young Chicks. Infect. Immun..

[13] Weiss G., Schaible U.E. (2015). Macrophage defense mechanisms against intracellular bacteria. Immunol. Rev..

[14] Medzhitov R., Janeway C. (2000). Innate immune recognition: Mechanisms and pathways. Immunol. Rev..

[15] Man S.M., Tourlomousis P., Hopkins L., Monie T.P., Fitzgerald K.A., Bryant C.E. (2013). *Salmonella* Infection Induces Recruitment of Caspase-8 to the Inflammasome To Modulate IL-1 beta Production. J. Immunol..

[16] Martinon F., Burns K., Tschopp J. (2002). The inflammasome: A molecular platform triggering activation of inflammatory caspases and processing of proIL-beta. Mol. Cell.

[17] Naseer N., Egan M.S., Reyes Ruiz V.M., Scott W.P., Hunter E.N., Demissie T., Rauch I., Brodsky I.E., Shin S. (2022). Human NAIP/NLRC4 and NLRP3 inflammasomes detect *Salmonella* type III secretion system activities to restrict intracellular bacterial replication. PLoS Pathog..

[18] Guo Y., Gu D., Huang T., Cao L., Zhu X., Zhou Y., Wang K., Kang X., Meng C., Jiao X. (2020). Essential role of *Salmonella* Enteritidis DNA adenine methylase in modulating inflammasome activation. BMC Microbiol..

[19] Fattinger S.A., Sellin M.E., Hardt W.-D. (2021). Epithelial inflammasomes in the defense against *Salmonella* gut infection. Curr. Opin. Microbiol..

[20] Jamilloux Y., Henry T. (2013). The inflammasomes: Platforms of innate immunity. Med. Sci..

[21] Sun Y.-H., Rolan H.G., Tsolis R.M. (2007). Injection of flagellin into the host cell cytosol by *Salmonella* enterica serotype Typhimurium. J. Biol. Chem..

[22] Miao E.A., Mao D.P., Yudkovsky N., Bonneau R., Lorang C.G., Warren S.E., Leaf I.A., Aderem A. (2010). Innate immune detection of the type III secretion apparatus through the NLRC4 inflammasome. Proc. Natl. Acad. Sci. USA.

[23] Ruiz V.M.R., Ramirez J., Naseer N., Palacio N.M., Siddarthan I.J., Yan B.M., Boyer M.A., Pensinger D.A., Sauer J.-D., Shin S. (2017). Broad detection of bacterial type III secretion system and flagellin proteins by the human NAIP/NLRC4 inflammasome. Proc. Natl. Acad. Sci. USA.

[24] Keller M., Rueegg A., Werner S., Beer H.-D. (2008). Active caspase-1 is a regulator of unconventional protein secretion. Cell.

[25] Ding J., Wang K., Liu W., She Y., Sun Q., Shi J., Sun H., Wang D.-C., Shao F. (2016). Pore-forming activity and structural autoinhibition of the gasdermin family. Nature.

[26] He W.T., Wan H., Hu L., Chen P., Wang X., Huang Z., Yang Z.H., Zhong C.Q., Han J. (2015). Gasdermin D is an executor of pyroptosis and required for interleukin-1β secretion. Cell Res..

[27] Miao E.A., Leaf I.A., Treuting P.M., Mao D.P., Dors M., Sarkar A., Warren S.E., Wewers M.D., Aderem A. (2010). Caspase-1-induced pyroptosis is an innate immune effector mechanism against intracellular bacteria. Nat. Immunol..

[28] Hapfelmeier S., Stecher B., Barthel M., Kremer M., Muller A.J., Heikenwalder M., Stallmach T., Hensel M., Pfeffer K., Akira S. (2005). The *Salmonella* pathogenicity island (SPI)-2 and SPI-1 type III secretion systems allow *Salmonella* serovar typhimurium to trigger colitis via MyD88-dependent and MyD88-independent mechanisms. J. Immunol..

[29] Qu Y., Misaghi S., Newton K., Maltzman A., Izrael-Tomasevic A., Arnott D., Dixit V.M. (2016). NLRP3 recruitment by NLRC4 during *Salmonella* infection. J. Exp. Med..

[30] Storek K.M., Monack D.M. (2015). Bacterial recognition pathways that lead to inflammasome activation. Immunol. Rev..

[31] Kaur J., Jain S.K. (2012). Role of antigens and virulence factors of *Salmonella enterica* serovar Typhi in its pathogenesis. Microbiol. Res..

[32] Keestra-Gounder A.M., Tsolis R.M., Baeumler A.J. (2015). Now you see me, now you don’t: The interaction of *Salmonella* with innate immune receptors. Nat. Rev. Microbiol..

[33] Hu G.-Q., Song P.-X., Chen W., Qi S., Yu S.-X., Du C.-T., Deng X.-M., Ouyang H.-S., Yang Y.-J. (2017). Cirtical role for *Salmonella* effector SopB in regulating inflammasome activation. Mol. Immunol..

[34] Hersh D., Monack D.M., Smith M.R., Ghori N., Falkow S., Zychlinsky A. (1999). The *Salmonella* invasin SipB induces macrophage apoptosis by binding to caspase-1. Proc. Natl. Acad. Sci. USA.

[35] Mueller A.J., Hoffmann C., Galle M., Van Den Broeke A., Heikenwalder M., Falter L., Misselwitz B., Kremer M., Beyaert R., Hardt W.-D. (2009). The S. Typhimurium Effector SopE Induces Caspase-1 Activation in Stromal Cells to Initiate Gut Inflammation. Cell Host Microbe.

[36] Bierschenk D., Monteleone M., Moghaddas F., Baker P.J., Masters S.L., Boucher D., Schroder K. (2019). The *Salmonella* pathogenicity island-2 subverts human NLRP3 and NLRC4 inflammasome responses. J. Leukoc. Biol..

[37] Miao E.A., Alpuche-Aranda C.M., Dors M., Clark A.E., Bader M.W., Miller S.I., Aderem A. (2006). Cytoplasmic flagellin activates caspase-1 and secretion of interleukin 1β via Ipaf. Nat. Immunol..

[38] Winter S.E., Winter M.G., Atluri V., Poon V., Romao E.L., Tsolis R.M., Baeumler A.J. (2015). The Flagellar Regulator TviA Reduces Pyroptosis by *Salmonella* enterica Serovar Typhi. Infect. Immun..

[39] Francis M.S., Amer A.A., Milton D.L., Costa T.R. (2017). Site-Directed Mutagenesis and Its Application in Studying the Interactions of T3S Components. Methods Mol. Biol..

[40] O’Toole G.A., Kolter R. (1998). Initiation of biofilm formation in Pseudomonas fluorescens WCS365 proceeds via multiple, convergent signalling pathways: A genetic analysis. Mol. Microbiol..

[41] Assouvie A., Daley-Bauer L.P., Rousselet G. (2018). Growing Murine Bone Marrow-Derived Macrophages. Methods Mol. Biol..

[42] Tang Y., Li F., Gu D., Wang W., Huang J., Jiao X. (2021). Antimicrobial Effect and the Mechanism of Diallyl Trisulfide against *Campylobacter jejuni*. Antibiotics.

[43] Martin M. (2011). Cutadapt removes adapter sequences from high-throughput sequencing reads. EMBnet. J..

[44] Kim D., Paggi J.M., Park C., Bennett C., Salzberg S.L. (2019). Graph-based genome alignment and genotyping with HISAT2 and HISAT-genotype. Nat. Biotechnol..

[45] Anders S., Pyl P.T., Huber W. (2015). HTSeq—A Python framework to work with high-throughput sequencing data. Bioinformatics.

[46] Love M.I., Huber W., Anders S. (2014). Moderated estimation of fold change and dispersion for RNA-seq data with DESeq2. Genome Biol..

[47] Malo N., Hanley J.A., Cerquozzi S., Pelletier J., Nadon R. (2006). Statistical practice in high-throughput screening data analysis. Nat. Biotechnol..

[48] Broz P., Newton K., Lamkanfi M., Mariathasan S., Dixit V.M., Monack D.M. (2010). Redundant roles for inflammasome receptors NLRP3 and NLRC4 in host defense against *Salmonella*. J. Exp. Med..

[49] Bao H., Wang S., Zhao J.-H., Liu S.L. (2020). *Salmonella* secretion systems: Differential roles in pathogen-host interactions. Microbiol. Res..

[50] Shin S., Brodsky I.E. (2015). The inflammasome: Learning from bacterial evasion strategies. Semin. Immunol..

[51] Philip N.H., Zwack E.E., Brodsky I.E. (2016). Activation and Evasion of Inflammasomes by *Yersinia*. Curr. Top Microbiol. Immunol..

[52] Bierschenk D., Boucher D., Schroder K. (2017). *Salmonella*-induced inflammasome activation in humans. Mol. Immunol..

[53] Fernandez De Henestrosa A.R., Badiola I., Saco M., Perez De Rozas A.M., Campoy S., Barbe J. (1997). Importance of the *galE* gene on the virulence of Pasteurella multocida. FEMS Microbiol. Lett..

[54] Fry B.N., Feng S., Chen Y.Y., Newell D.G., Coloe P.J., Korolik V. (2000). The *galE* gene of *Campylobacter jejuni* is involved in lipopolysaccharide synthesis and virulence. Infect. Immun..

[55] Husseiny M.I., Hensel M. (2008). Construction of highly attenuated *Salmonella enterica* serovar Typhimurium live vectors for delivering heterologous antigens by chromosomal integration. Microbiol. Res..

[56] Gilman R.H., Hornick R.B., Woodard W.E., DuPont H.L., Snyder M.J., Levine M.M., Libonati J.P. (1977). Evaluation of a UDP-glucose-4-epimeraseless mutant of *Salmonella* typhi as a liver oral vaccine. J. Infect. Dis..

[57] Clare B. (2021). Inflammasome activation by *Salmonella*. Curr. Opin. Microbiol..

[58] Miao E.A., Rajan J.V. (2011). *Salmonella* and Caspase-1: A complex interplay of detection and evasion. Front Microbiol..

[59] Liu Q., Zhang D., Hu D., Zhou X., Zhou Y. (2018). The role of mitochondria in NLRP3 inflammasome activation. Mol. Immunol..

[60] Lawley T.D., Chan K., Thompson L.J., Kim C.C., Govoni G.R., Monack D.M. (2006). Genome-wide screen for *Salmonella* genes required for long-term systemic infection of the mouse. PLoS Pathog..

[61] Wynosky-Dolfi M.A., Snyder A.G., Philip N.H., Doonan P.J., Poffenberger M.C., Avizonis D., Zwack E.E., Riblett A.M., Hu B., Strowig T. (2014). Oxidative metabolism enables *Salmonella* evasion of the NLRP3 inflammasome. J. Exp. Med..

[62] Sanman L.E., Qian Y., Eisle N.A., Ng T.M., van der Linden W.A., Monack D.M., Weerapana E., Bogyo M. (2016). Disruption of glycolytic flux is a signal for inflammasome signaling and pyroptotic cell death. eLife.

[63] Nnalue N.A., Stocker B.A. (1986). Some *galE* mutants of *Salmonella* choleraesuis retain virulence. Infect. Immun..

[64] Eisele N.A., Ruby T., Jacobson A., Manzanillo P.S., Cox J.S., Lam L., Mukundan L., Chawla A., Monack D.M. (2013). *Salmonella* require the fatty acid regulator PPARδ for the establishment of a metabolic environment essential for long-term persistence. Cell Host Microbe.

[65] Giblin J.P., Hewlett L.J., Hannah M.J. (2008). Basal secretion of von Willebrand factor from human endothelial cells. Blood.

[66] He M., Gu J., Zhu J., Wang X., Wang C., Duan C., Ni Y., Lu X., Li J. (2018). Up-regulation of Dyrk1b promote astrocyte activation following lipopolysaccharide-induced neuroinflammation. Neuropeptides.

[67] Gervais O., Ueno K., Kawai Y., Hitomi Y., Aiba Y., Ueta M., Nakamura M., Tokunaga K., Nagasaki M. (2021). Regional heritability mapping identifies several novel loci (STAT4, ULK4, and KCNH5) for primary biliary cholangitis in the Japanese population. Eur. J. Hum. Genet..

[68] Wang M., Jing J., Li H., Liu J., Yuan Y., Sun L. (2021). The expression characteristics and prognostic roles of autophagy-related genes in gastric cancer. PeerJ.

[69] Biasizzo M., Kopitar-Jerala N. (2020). Interplay between NLRP3 inflammasome and autophagy. Front. Immunol..

[70] Iyer S.S., Cheng G. (2012). Role of interleukin 10 transcriptional regulation in inflammation and autoimmune disease. Crit. Rev. Immunol..

[71] Sun Y., Ma J., Li D., Li P., Zhou X., Li Y., He Z., Qin L., Liang L., Luo X. (2019). Interleukin-10 inhibits interleukin-1β production and inflammasome activation of microglia in epileptic seizures. J. Neuroinflamm..

[72] Jiao Y., Xia Z., Zhou X., Guo Y., Guo R., Kang X., Wu K., Sun J., Xu X., Jiao X. (2018). Signature-tagged mutagenesis screening revealed the role of lipopolysaccharide biosynthesis gene rfbH in smooth-to-rough transition in *Salmonella* Enteritidis. Microbiol. Res..

[73] Chiang S.L., Rubin E.J. (2002). Construction of a mariner-based transposon for epitope-tagging and genomic targeting. Gene.

[74] Wang S.Y., Lauritz J., Jass J., Milton D.L. (2002). A ToxR homolog from Vibrio anguillarum serotype O1 regulates its own production, bile resistance, and biofilm formation. J. Bacteriol..

[75] Guzman L.M., Belin D., Carson M.J., Beckwith J. (1995). Tight regulation, modulation, and high-level expression by vectors containing the arabinose PBAD promoter. J. Bacteriol..

[76] Charpentier X., Oswald E. (2004). Identification of the secretion and translocation domain of the enteropathogenic and enterohemorrhagic Escherichia coli effector Cif, using TEM-1 beta-lactamase as a new fluorescence-based reporter. J. Bacteriol..

