# Supplementary material for: Salmonella Enteritidis GalE Protein Inhibits LPS-Induced NLRP3 Inflammasome Activation
Source: Microorganisms. 2022 Apr 26;10(5):911. doi: 10.3390/microorganisms10050911 (PMC9145252; doi:10.3390/microorganisms10050911)
Supplement: Supplementary file 1 [file microorganisms-10-00911-s001.zip › microorganisms-1695865-supplementary.pdf]

Table S1 Bacterial strains and plasmids used in this study

Strain	Description	Source
Escherichia coli		
X7213λpir	Host for π requiring plasmids, conjugal donor	[1]
X7213λpir-pSC189	X7213 λ*pir* with pSC189, Km^r, Cm^r	This study
***Salmonella* Enteritidis**		
C50336	Wild type	[2]
C50336Δ*fliC*	C50336, In-frame deletion in *fliC* (Parent strain)	This study
Δ*fliC*Δ*galE*	Δ*fliC*, In-frame deletion in *galE*	This study
Δ*fliC*Δ*galE*::*galE*	Δ*fliC*Δ*galE* with pBAD33 expressing the *galE*, Cm^r	This study
Δ*fliC*Δ*galE*::vector	Δ*fliC*Δ*galE* with pBAD33, Cm^r	This study
Δ*fliC*-pCX340	Δ*fliC*Δ with pCX340, Tet^r	This study
Δ*fliC*-pCX340-*galE*	Δ*fliC*with pCX340-*galE* expressing the *genE*, Tet^r	This study
**Plasmids**		
pSC189	Transposon delivery vector, R6K, Km^r, Cm^r	[3]
pDM4	Suicide vector, *pir* dependent, R6K, *SacBR*, Cm^r	[4]
pBAD33	p15A-based expression vector utilizing P_{BAD} promoter, Cm^r	[5]
pBAD33-*galE*	pBAD33 derivative containing *galE*, Cm^r	This study
pCX340	pBR322 derivative, used to fuse effectors to TEM-1-β-lactamase, Tet^r	[6]
pCX340-*galE*	pCX340 derivative containing *galE*, Cm^r	This study

1. Jiao, Y.; Xia, Z.; Zhou, X.; Guo, Y.; Guo, R.; Kang, X.; Wu, K.; Sun, J.; Xu, X.; Jiao, X.; Pan, Z.; Liu, X. Signature-tagged mutagenesis screening revealed the role of lipopolysaccharide biosynthesis gene *rfbH* in smooth-to-rough transition in *Salmonella* Enteritidis. *Microbiol Res* **2018**, *212-213*, 75-79. doi:10.1016/j.micres.2018.05.001.
2. Guo, Y.; Gu, D.; Huang, T.; Cao, L.; Zhu, X.; Zhou, Y.; Wang, K.; Kang, X.; Meng, C.; Jiao, X.; Pan, Z. Essential role of *Salmonella* Enteritidis DNA adenine methylase in modulating inflammasome activation. *BMC Microbiol* **2020**, *20*, 226. doi:10.1186/s12866-020-01919-z.
3. Chiang, S.L.; Rubin, E.J. Construction of a mariner-based transposon for epitope-tagging and genomic targeting. *Gene* **2002**, *296*, 179-185. doi:10.1016/s0378-1119(02)00856-9.
4. Wang, S.Y.; Lauritz, J.; Jass, J.; Milton, D.L. A ToxR homolog from Vibrio anguillarum serotype O1 regulates its own production, bile resistance, and biofilm formation. *J. Bacteriol.* **2002**, *184*, 1630-1639. doi:10.1128/jb.184.6.1630-1639.2002.
5. Guzman, L.M.; Belin, D.; Carson, M.J.; Beckwith, J. Tight regulation, modulation, and high-level expression by vectors containing the arabinose PBAD promoter. *J. Bacteriol.* **1995**, *177*, 4121-4130. doi:10.1128/jb.177.14.4121-4130.1995.
6. Charpentier, X.; Oswald, E. Identification of the secretion and translocation domain of the enteropathogenic and enterohemorrhagic *Escherichia coli* effector Cif, using TEM-1 beta-lactamase as a new fluorescence-based reporter. *J. Bacteriol.* **2004**. *186*, 5486-5495, doi:10.1128/JB.186.16.5486-5495.2004.

Table S2 The primers used in this study

Primer name	Primer sequence (5′ to 3′)	Target
AB1	GGCCACGCGTCGACTAGTACNNNNNNNNNNACGCC	Transposon identify
AB2	GGCCACGCGTCGACTAGTACNNNNNNNNNNCCTGG	Transposon identify
AB3	GGCCACGCGTCGACTAGTACNNNNNNNNNNCCTCG	Transposon identify
ABS	GGCCACGCGTCGACTAGTAC	Transposon identify
SP1	GCTGACCGCTTCCTCGTGCTTTACG	Transposon identify
SP2	CATCGCCTTCTATCGCCTTCTTGAC	Transposon identify
pSC189-seq	CGCGAAGTTCCTATTCCGAAGTTCC	Transposon identify
pDM4-F	GGTGCTCCAGTGGCTTCTGTTTCTA	Deletion mutant
pDM4-R	CAGCAACTTAAATAGCCTCTAAT	Deletion mutant
galE-up-F	GAGCGGATAACAATTTGTGGAATCCCGGGATGAATG AACAAGGCGTGAACTACAA	Deletion mutant
galE-up-R	AACGTCCTTACATAATTCGCTCCGTTAAGCCTATG	Deletion mutant
galE-down-F	GCGAATTATGTAAGGACGTTGTTATGACCCCATTT	Deletion mutant
galE-down-R	AGCGGAGTGTATATCAAGCTTATCGATACCGCTCG GCGAAATAGGCTTTTTGTAA	Deletion mutant
galE-in-F	AGGTGGTAGCGGTTACATTGGAAGT	Deletion mutant
galE-in-R	TACTAAAGGCGTTGACCACATCCAG	Deletion mutant
galE-out-F	GAAGGATTTACCTGGATACCCGCAA	Deletion mutant
galE-out-R	GACATAATCCACCAGCATTGGCGAG	Deletion mutant
pBAD33-F	TCTACTGTTTCTCCATACCCGTTTT	Complementary
pBAD33-R	TTCTGCGTTCTGATTTAATCTGTAT	Complementary
pBAD33-*galE*-F	GGGCTAGCGAATTCGAGCTCGGTACCTAAAGGAAGACGTTATGAG AGTATTGGTTACAGGTGGTA	Complementary
pBAD33-*galE*-R	TCTCATCCGCCAAAACAGCCAAGCTTTAGTGATGATGATGATGATG ATCTGGGTATCCCTGCGGATGG	Complementary
pCX340-F	AGACAATCTGTGTGGGCACTCGACC	FRET
pCX340-R	TTCTGAGAATAGTGTATGCGGCGAC	FRET
pCX340-*galE*-F	AAGGAGGAATAACATATGATGAGAGTATTGGTTACAGGTGGTAG	FRET
pCX340-*galE*-R	GTGCGAATTCTCCGCGGAGGTACCATCTGGGTATCCCTGCGGATGG	FRET

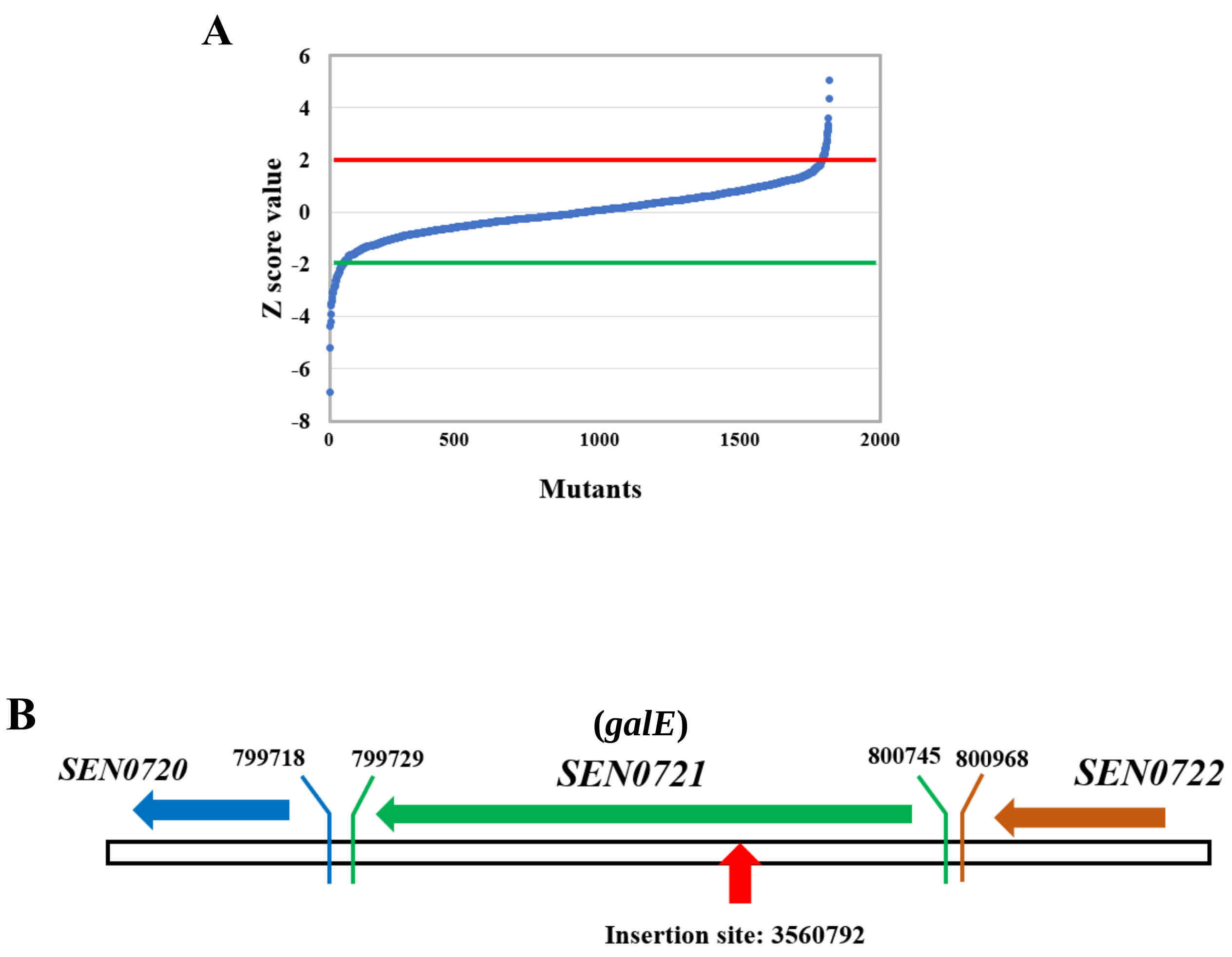
A
6
4
2
0
-2
-4
-6
-8
Z score value
0
500
1000
1500
2000
Mutants
B
(galE)
SEN0720
799718
799729
SEN0721
800745
800968
SEN0722
Insertion site: 3560792

Figure S1

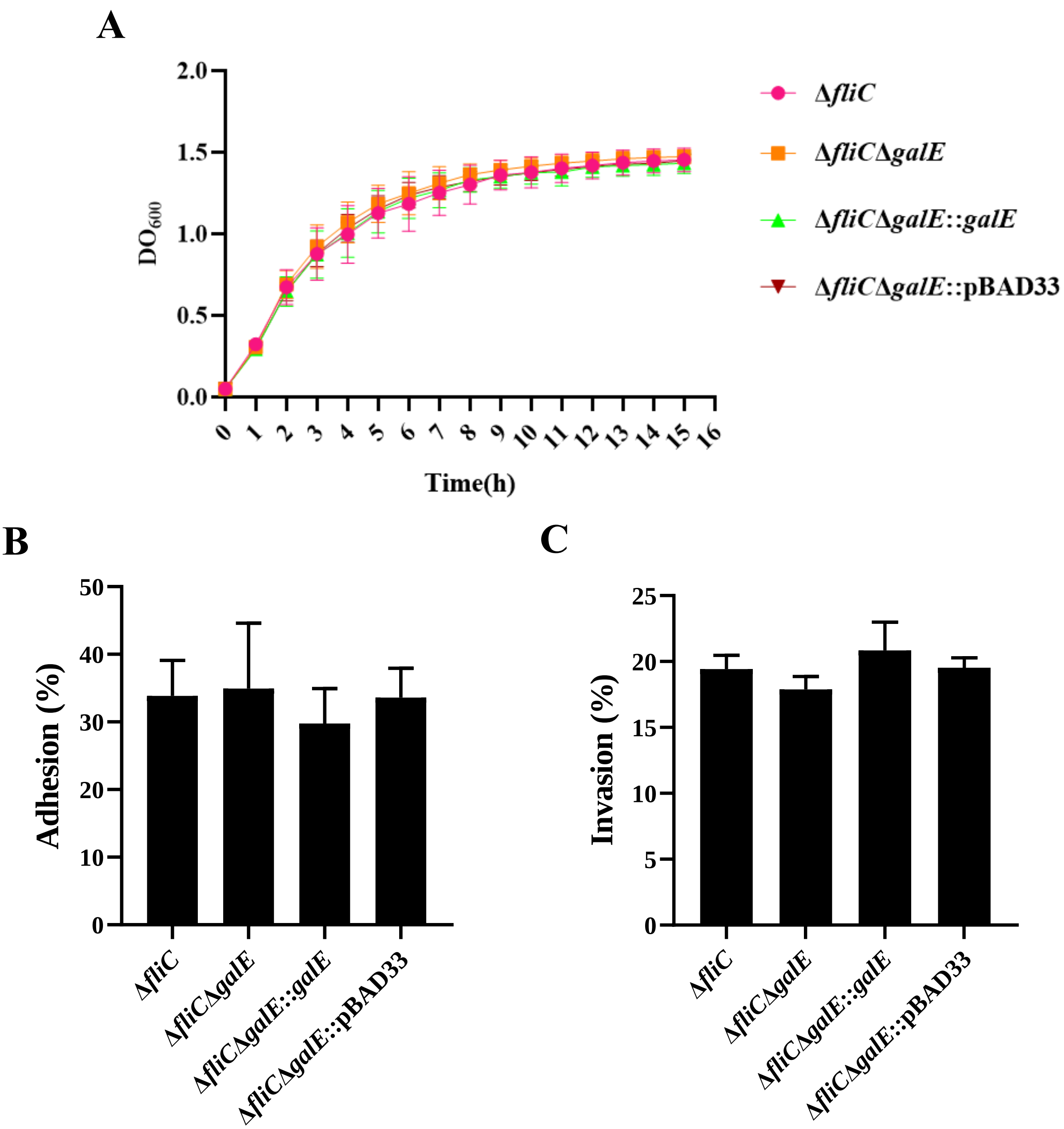
A
2.0
1.5
1.0
0.5
0.0
DO600
0 1 2 3 4 5 6 7 8 9 10 11 12 13 14 15 16
Time(h)
ΔfliC
ΔfliCΔgalE
ΔfliCΔgalE::galE
ΔfliCΔgalE::pBAD33
B
50
40
30
20
10
0
Adhesion (%)
ΔfliC
ΔfliCΔgalE
ΔfliCΔgalE::galE
ΔfliCΔgalE::pBAD33
C
25
20
15
10
5
0
Invasion (%)
ΔfliC
ΔfliCΔgalE
ΔfliCΔgalE::galE
ΔfliCΔgalE::pBAD33

Figure S2
